# Theory of neural communication based on spatio-temporal coding

**DOI:** 10.1186/1471-2202-12-S1-P38

**Published:** 2011-07-18

**Authors:** Myoung Won Cho, Moo Young Choi

**Affiliations:** 1 Korea Institute for Advanced Study, Seoul 130-722, Korea; 2 Department of Physics and Astronomy and Center for Theoretical Physics, Seoul National University, Seoul 151-747, Korea

## 

Pattern coding is a general concept for neural coding, which indicates that the objective meaning of information can be represented by spatio-temporal firing patterns of a group of neurons [1]. We introduce a feasible way through which spatio-temporal firing patterns represent complex information systematically. Provided that neural codes represent features in a vector space, neural communication channels can be characterized by independent pattern components constituting basis functions in the Hilbert space. Specific applied forms of the method, depending on the choice of basis functions, reduce to the traditional coding schemes, including rate coding, temporal coding, correlation coding, independent-spike coding, population coding, phase coding, and so on. In addition, it is suggested that the ordinary neural code in the brain might take a more elaborate form, in such a way that neural networks can send or receive complex information effectively and robustly through mutable spike trains. We discuss corresponding statistics of neural codes based on the theory. Finally, we present the scheme of a cortical module as the processing unit of communication and computation based on spatio-temporal coding. It may also be applied to modelling the stimulus-response of cortical neurons.

## Conclusion

We present a theory as to how neural modules communicate with each other effectively via spatio-temporal firing patterns, and propose, based on the theory, how to phrase neural codes from observed firing patterns and how to model the stimulus-response relationship for cortical neurons.
